# High Levels of Microplastics in the Arctic Sea Ice Alga *Melosira arctica*, a Vector to Ice-Associated and Benthic Food Webs

**DOI:** 10.1021/acs.est.2c08010

**Published:** 2023-04-21

**Authors:** Melanie Bergmann, Steve Allen, Thomas Krumpen, Deonie Allen

**Affiliations:** † HGF-MPG Group for Deep Sea Ecology and Technology, Alfred-Wegener-Institut Helmholtz-Zentrum für Polar- und Meeresforschung, 27570 Bremerhaven, Germany; ‡ Ocean Frontiers Institute, Dalhousie University, B3H 4R2 Nova Scotia, Canada; § Sea Ice Physics, Alfred-Wegener-Institut Helmholtz-Zentrum für Polar- und Meeresforschung, 27570 Bremerhaven, Germany; ∥ School of Geography, Earth and Environmental Science, University of Birmingham, B15 2TT Birmingham, U. K.; ⊥ School of Physical and Chemical Sciences, University of Canterbury, 8041 Christchurch, New Zealand

**Keywords:** Arctic, ballasting, Fram Strait, sea ice, ice algae, Melosira arctica, microplastic, plastic, sympagic, polar regions

## Abstract

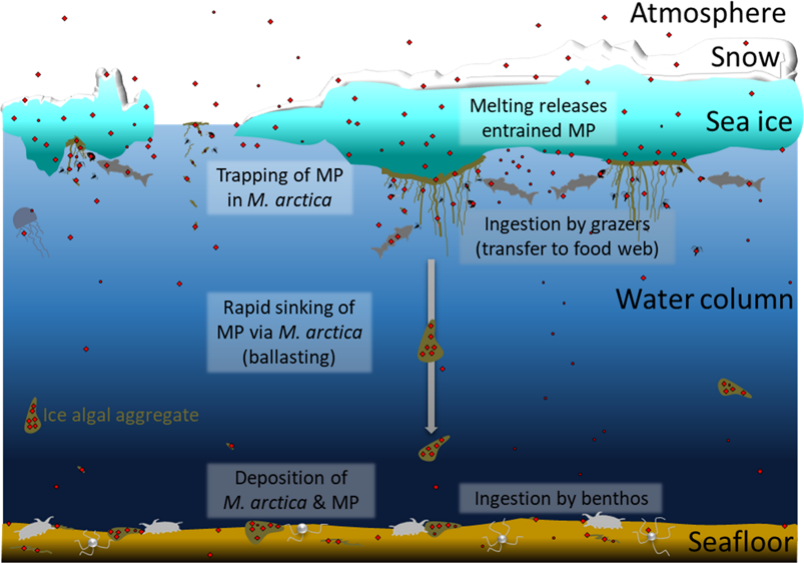

Plastic pollution has become ubiquitous with very high quantities detected even in ecosystems as remote as Arctic sea ice and deep-sea sediments. Ice algae growing underneath sea ice are released upon melting and can form fast-sinking aggregates. In this pilot study, we sampled and analyzed the ice algae*Melosira arctica*and ambient sea water from three locations in the Fram Strait to assess their microplastic content and potential as a temporary sink and pathway to the deep seafloor. Analysis by μ-Raman and fluorescence microscopy detected microplastics (≥2.2 μm) in all samples at concentrations ranging from 1.3 to 5.7 × 10^4^ microplastics (MP) m^–3^ in ice algae and from 1.4 to 4.5 × 10^3^ MP m^–3^ in sea water, indicating magnitude higher concentrations in algae. On average, 94% of the total microplastic particles were identified as 10 μm or smaller in size and comprised 16 polymer types without a clear dominance. The high concentrations of microplastics found in our pilot study suggest that*M. arctica* could trap microplastics from melting ice and ambient sea water. The algae appear to be a temporary sink and could act as a key vector to food webs near the sea surface and on the deep seafloor, to which its fast-sinking aggregates could facilitate an important mechanism of transport.

## Abstract 2

This study shows high microplastic concentrations in Arctic ice algae relative to ambient sea water, indicating that it could be a temporary sink and vector to ice-associated and benthic food webs.

## Introduction

Since the 1970s, plastic production has increased annually by 8% and could double in the coming 20 years.^1^ Since there is still no solution for the end of life of most plastic products, plastic pollution has spread to ecosystems around the globe.^2^ Under the influence of light, mechanical abrasion, and temperature fluctuations, plastic items break down into ever smaller fragments. Particles ≤5 mm are termed microplastics (MPs) and considered irretrievable.^3^ Despite the remoteness of the Arctic, MPs have become particularly abundant and ubiquitous in Nordic ecosystems,^4,5^ including Arctic snow, glaciers, sea water, and very high concentrations in deep-sea sediments and sea ice.^6−10^ Not surprisingly, MPs were also reported in organisms associated with sea ice such as sympagic zooplankton^11^ and polar cod (*Boreogadus saida*).^12,13^

**Figure 1:**
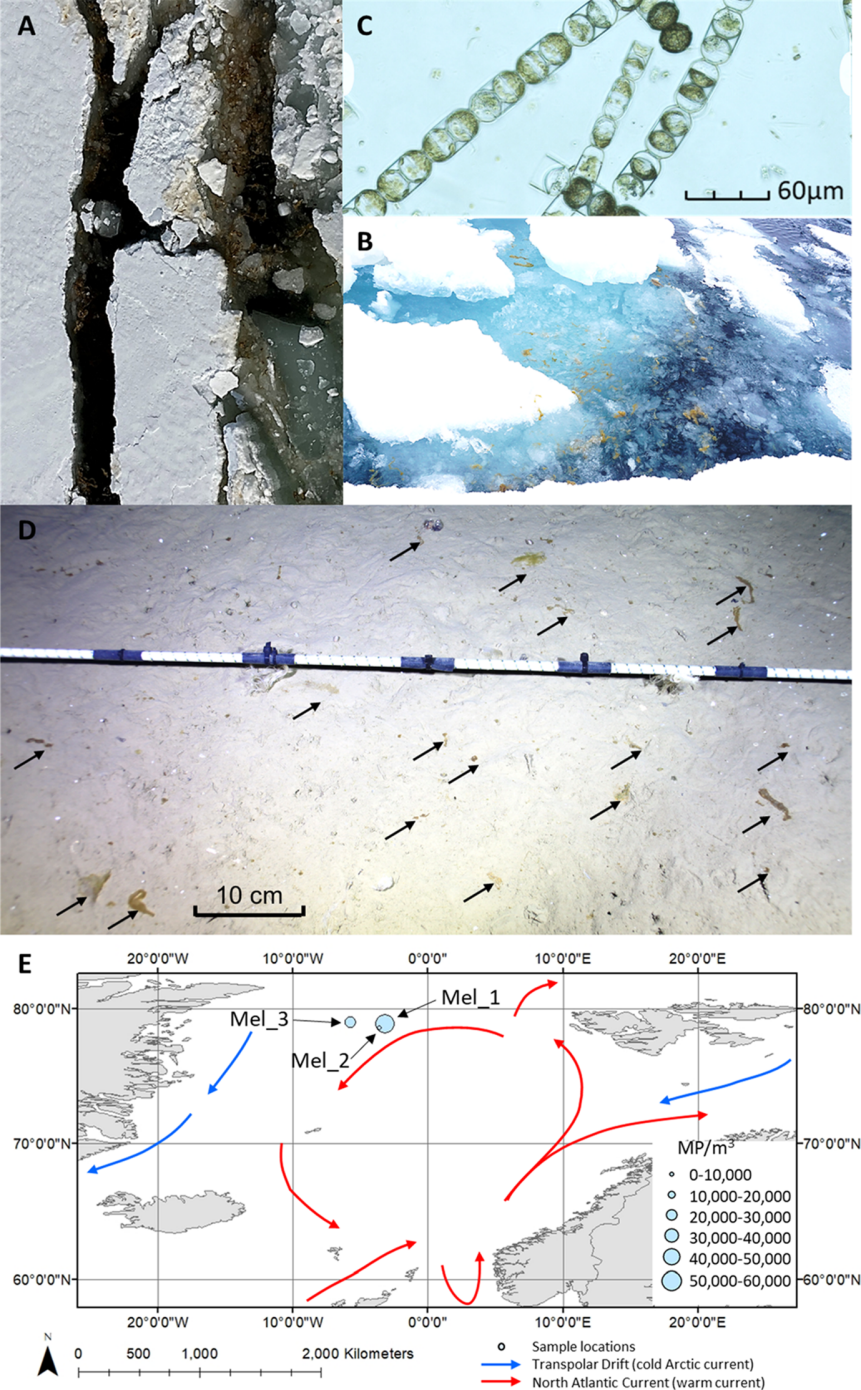
(A) Photographs of *M. arctica* in sea water and broken ice surrounding the ice floes at sites Mel_2 and (B) Mel_3. (C) Microscopic photograph of a subsample of Mel_1 taken for the species identification of *M. arctica* (credit with permission: A. Kraberg, AWI, magnification: ×400). (D) Ice algae (black arrows) deposited on the seafloor at ∼2500 m depth in the Fram Strait, depicted by a time-lapse camera on 24/07/20 (credit with permission: F. Wenzhöfer, AWI). (E) Map of the study area and sampling sites in the Fram Strait; red arrows indicate water masses of the Atlantic inflow, and blue arrows depict cooler waters of polar origin (current arrows are inspired with permission by Macdonald et al.^21^).

The centric diatom *Melosira arctica* (Figure 1) can be considered a keystone to Arctic ecosystems feeding a variety of zooplankton species near the sea surface.^14^ While its extent and distribution are still poorly known,^15^ it is an important primary producer, which accounted for 45% of the Arctic primary production in 2012,^16^ the year of the lowest hitherto recorded Arctic sea ice extent. This unicellular microalga forms colonial filamentous strands, nets, ropes, mats, and curtains of up to 3 m length underneath annual and multi-year sea ice and is characterized by a patchy distribution.^15,17,18^ During sea ice melting, *M. arctica* detaches from the sea ice, and its aggregates float freely^15,19^ or sink rapidly to the ocean floor (Figure 1A–C), feeding epibenthic organisms such as brittlestars and sea cucumbers.^16,17,20^*M. arctica* is widely distributed with reports from the Canada Basin, Chukchi Sea, Nunavut, northern Baffin Bay, Arctic Ocean, Central Arctic, Laptev Sea, northeast Greenland, and the Barents Sea.^15−17,20^

Given its sticky filamentous morphology and its close association with Arctic sea ice, which carries very high levels of MPs, we hypothesize that *M. arctica* entraps MPs released from the overlying melting sea ice and surrounding sea water. To test this hypothesis, we analyzed *M. arctica* samples that were taken opportunistically during helicopter-based ice stations of a research campaign in 2021 at the HAUSGARTEN observatory in the Fram Strait. Satellite-based backtracking of the sampled ice floes was undertaken to pinpoint the origin and trajectory of the ice floes and potentially also of the entrained MPs. It is important to fill this knowledge gap since*M. arctica* could function as a vector of MPs into sub-ice and benthic food webs. Given the importance of*M. arctica*in Arctic benthic food webs, it is important that we understand the likelihood of *M. arctica* acting as a scavenger and transport vector for MP and plastic chemicals.

## Materials and Methods

### Sampling Procedure

Three *M. arctica* samples were collected in the Arctic circle in 2021 during expedition PS121 of the research icebreaker RV Polarstern to the HAUSGARTEN observatory, which saw substantial quantities of ice floes. Samples were collected from sea water adjacent to the floating sea ice (Table 1, Figure 1E).

**Table 1:** Details of *M. arctica* and Sea Water Sampling

station	time (UTC)	date	position latitude	position longitude	ice salinity (EC mS cm^–1^)	ice floe thickness (m)
Mel_1	13:00 h	13/06/21	78.90194 N	3.151111 W	3.78–35.4	2.13
Mel_2	15:00 h	14/06/21	78.59433 N	3.548667 W	3.38–23.6	2.02
Mel_3	13:00 h	15/06/21	79.02889 N	5.713889 W	14.26–23.5	1.52

Ice floes were visited by a helicopter (D-HAOE, D-HAPS, HeliService, Emden, Germany) during a sampling campaign for MPs in snow, melt ponds, sea water, and sea ice and had to be safe for landing. Here, we present only the results from *M. arctica* and corresponding sea water samples as all other analyses are still ongoing. Bending over the ice edge (secured by a safety rope), *M. arctica* aggregates that were free-floating among the sea ice (Figure 1A,B) were scooped from the sea water using a nylon net of ∼1 mm porosity and placed into triple MilliQ pre-rinsed 2 L stainless-steel containers (ECOtanka). Sea water samples were also grabbed from the sea surface with triple MilliQ pre-rinsed 2 L stainless-steel containers attached to a hemp rope (4 L in total). Field blank samples for each location were created using 2 L stainless-steel containers left open for the duration of sampling with 100 mL of MilliQ water in the base to retain deposited MP particles. Scientists stood downwind of the sampling location when taking the sample to minimize any clothing contamination since red survival suits and leather gloves had to be worn for work safety. The position of the ice floe was recorded at the end of the visit by a hand-held GPS device (Table 1).

### Sample Preparation

Back on the research vessel, ice algal samples were transferred into triple MilliQ-rinsed glass vials and refrigerated (at 4 °C) until analysis. A subsample was taken for species identification under the microscope (Figure 1C). The sea water samples were filtered onto Whatman QMA quartz filters (2.2 μm porosity, liquid) and stored at room temperature in sealed metal containers (20 °C) in the laboratory. Organic matter was digested from sea water filtered samples using hydrogen peroxide^7^ (40 mL of H_2_O_2_ was placed over the filtered samples held in borosilicate glass filtration equipment and incubated at room temperature for 48 h and then flushed with 200 mL of MilliQ). Then, the air-dried filter was stored in sealed metal containers until analysis. In a dedicated MP laboratory, *M. arctica* samples were subsampled for MP and nanoplastic (NP) analysis in a laminar flow fume hood that had been pre-cleaned to minimize contamination. Using sterilized stainless-steel tweezers and sterilized stainless-steel micro spatulas, subsamples of 3–7 g of wet material (see Supplementary Data) were placed into sterilized borosilicate glass test tubes (four subsamples per site). 20 mL of 30% w/w hydrogen peroxide (H_2_O_2_, pre-filtered through a 0.2 μm aluminum oxide filter) was added to each sample with sterilized aluminum foil lids, and then, the samples were placed in a dry heat block for 48 h at 50 °C. An additional 10 mL of H_2_O_2_ was then added, and the samples were returned to the dry heat block for another 48 h at 50 °C. The samples were then filtered onto 47 mm diameter aluminum oxide filters (0.2 μm porosity) and flushed with 250 mL of MilliQ for Raman analysis. Filters did not become blind during any of the collection or filtration processes. The filters were stored in sterilized 50 mm diameter aluminum containers to minimize contamination.

### Raman Analysis

All samples were then analyzed by μ-Raman microscopy following published protocols developed by Zhao et al.^22^ and used consistently by Allen et al.^23−25^ (Horiba Xplora Plus) using a 785 nm laser (spatial resolution of 1 μm), 1200 gr mm^–1^ grating, 50 μm slit, and 25% power (filter), with spectra collected over 200–2000 cm^–1^ Raman shift using five acquisitions of 10 s. 25% of each filter was assessed following the filter area representation of Huppertsberg and Knepper.^26^ Raman spectra were analyzed using Spectragryph,^27^ SloPP, SloPP-E,^28^ and siMPle^29^ Raman reference libraries supplemented with in-house plastic spectra references. Spectra with a ≥80% hit rate were counted as plastic particles and included in the total sample MP count.^23,24,30−33^ The limit of quantification (LOQ, s. below) for this analysis was set to 1.2 μm for *M. arctica* samples and 2.2 μm for sea water samples. This was due to the different porosity of filtration possible for the two different matrices. The μ-Raman results were used to quantify the number of MP particles and the quantity of different plastic polymer types in each sample according to Armbruster and Pry^34^ and Dawson et al.,^35^ where LOQ ≥ LOD (Limit of Detection) and LOD = (mean_blank_ + 1.645 (SD_blank_)) + 1.645 (SD_lowest sample result_).

### Fluorescence Microscopy

Samples were secondarily analyzed using Nile Red fluorescence microscopy^36−38^ to provide a particle count check and the particle shape and size representative of each sample. Particles were identified as fibers (1:3 width-to-length ratio)^39,40^ or fragments due to the uncertainty in defining films. Samples were stained with Nile Red powder (Sigma Aldrich) dissolved in methanol (99% pure, Sigma Aldrich) to result in 0.1 μg mL^–1^ solution and pre-filtered through sterilized glass fiber filters (Whatman GF/C, 1.2 μm porosity).^37^ The Nile Red solution was stored in a sterilized, sealed glass container covered in aluminum foil in the dark at 4 °C. Stained samples were oven-dried (30 °C) overnight and then placed in the fridge (4 °C) in sealed aluminum containers to rest prior to fluorescence microscopy imaging. A Nikon LV100ND fluorescence microscope with an FITC (Fluorescein) filter was used to image 25% of each filter following the filter area representation.^26^ Fluorescent particle dimensions (including circularity and the ferret diameter) and counts were collected using FIJI software^37,41^ following published standards.^41,42^ Nile Red fluorescence MP counts were used to confirm the Raman MP quantification and to obtain particle size and shape information.

### Blanks, Positive Controls, and Contamination Mitigation

Sample preparation was undertaken in a dedicated MP laboratory with controlled access, a dedicated HEPA filter, and a laminar flow fume hood. Prior to sample preparation, the laboratory and laboratory fume hood were pre-cleaned to minimize laboratory contamination. Pre-cleaning was completed using a plastic-free cleaning fluid followed by MilliQ water and clean 100% cotton lint-free clothes. Only cotton clothing and cotton lab coats were worn in the laboratory, nitrile gloves were used for all chemical handling, and all materials were sterilized (superheated to 450 °C) or triple-washed with MilliQ (as appropriate) between each use.

Field blank samples underwent the sample digestion process and full analytical process, resulting in all field blanks being full process blanks. All samples were blank-corrected using these field blank μ-Raman results. Blanks had on average 5 (± 2) particles per sampled filter area (<20% of the analyzed field MP particle results per total filter count and therefore supporting field result validation). Positive controls (laboratory blanks spiked with known plastic particles) were also created to check the full process recovery efficiency. A spiked solution was made from polyethylene particles ranging from 2 to 110 μm (particle size distribution described using a Mastersizer 2000), and spiked solution samples underwent the full preparation and analysis process as field samples and field blanks. The μ-Raman analytical efficiency (the effective polymer counts relative to this analytical method quantified by positive controls) was calculated to be 89% (± 12%) and the Nile Red fluorescence microscopy analysis efficiency to be 86% (± 12%) (see the Supplementary Information). It is acknowledged that spiked recovery samples should ideally include a range of polymer types, and future studies will incorporate this advancement.

### Backtracking of Ice Floes Surrounding Ice Algae

The possible origin and pathways of the ice algal samples were determined using two different satellite-based tracking approaches: in the Fram Strait, floe locations were tracked visually using optical satellite data. Visual identification and tracking were carried out using NASA’s interactive interface to display full-resolution satellite images on a daily basis (https://worldview.earthdata.nasa.gov/). After the floes reached the closed ice cover at ∼82°N, tracking was continued using an automated backtracking algorithm based on passive microwave data. Note that the automated tracking is less accurate in the Fram Strait,^43^ which is why we have limited its use to the central Arctic. The applied automated backtracking algorithm, products, and their accuracy are described in Krumpen et al.^44^ Tracking was discontinued if the ice concentration at a specific location along the backward trajectory dropped below 20% and we assumed the ice to be formed.

### Data Analysis

Nile Red fluorescence microscopy analysis was undertaken using FIJI(ImageJ)^37,41^ exported into standard Excel software for blank correction and result plotting. Similarly, Raman spectral analysis was undertaken using Spectragryph^27^ software with the results exported into standard Excel software for blank correction and result plotting. Blank correction was undertaken by the particle size (for particle size distribution analysis) and the polymer type (for polymer sample composition analysis). Blank correction of all samples was undertaken using the full process field blank sample results to ensure that all field and laboratory actions were accounted for in contamination assessment and the most conservative approach to blank correction was used. Simple correlation and statistical tests were completed using Excel. The underlying data and test results are available in the Supplementary Information.

## Results and Discussion

**Figure 2:**
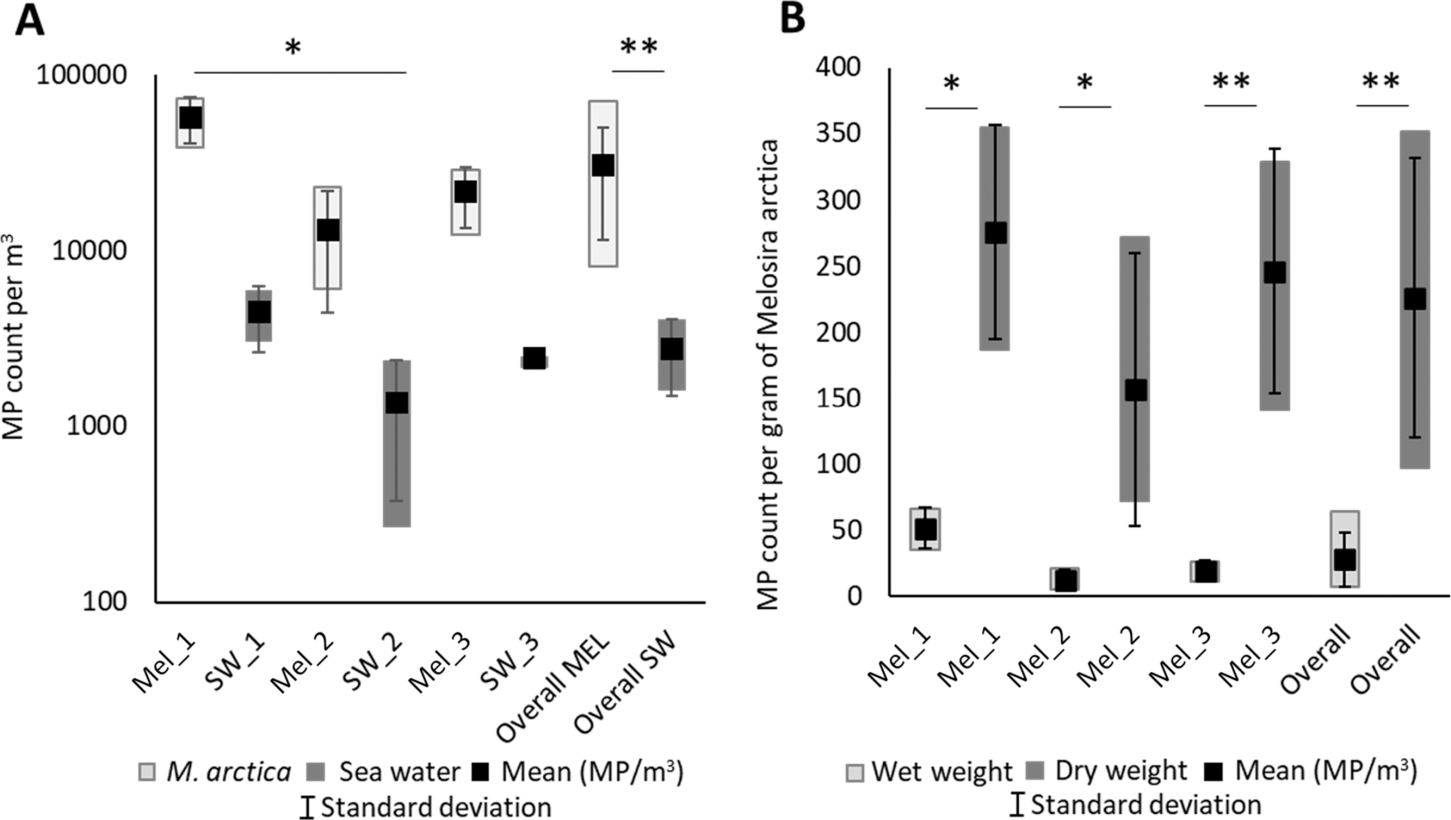
MP quantities, particle size distribution, and polymer types detected in the samples of the ice alga *M. arctica*. (A) Boxplot of MP particle counts (10th–90th percentile), means, and standard deviations in *M. arctica* and surrounding sea water samples (MP m^–3^). (B) *M. arctica* MP counts (10th–90th percentile), means, and standard deviations relative to the sample wet and dry weights (g). The asterisks indicate datasets that present a significant difference (* = *p* < 0.05, ** = *p* < 0.01).

MP particles were found in all subsamples across a variety of polymer types and particle sizes. A total of 400 MP particles were identified from the 12 subsamples (Figure 2A and Supplementary Information). *M. arctica* samples contained 5–66 MP particles per mg wet weight (Figure 2B), on average 28 (±20 standard deviation, σ) MP particles per sample, and an estimated equivalent mean of 3.1 ± 1.9 × 10^4^ MP m^–3^ (see Table S1 for sample MP counts, weights, and volumes and resulting MP values per mg and m^3^). While sea ice in this region contained higher MP levels (1.2 × 10^7^–1.1 × 10^6^ MP m^–3^),^7^ sea ice concentrates suspended organic carbon by two orders of magnitude relative to ambient sea water during ice formation through a process termed “scavenging”, which partly explains the exceptionally high MP concentrations (suspended organic carbon in Arctic sea ice may also be two orders of magnitude greater than that in sea water).^45^ If two orders of magnitude are deducted from the MP concentrations for a better comparison, the MP concentrations of sea ice and ice algae become strikingly similar at the magnitude scale despite differences in the methodology used. MP concentrations in water samples ranged from 4.5 to 1.4 × 10^3^ MP m^–3^ with an overall mean of 2.8 ± 1.3 × 10^3^ MP m^–3^.

**Figure 3:**
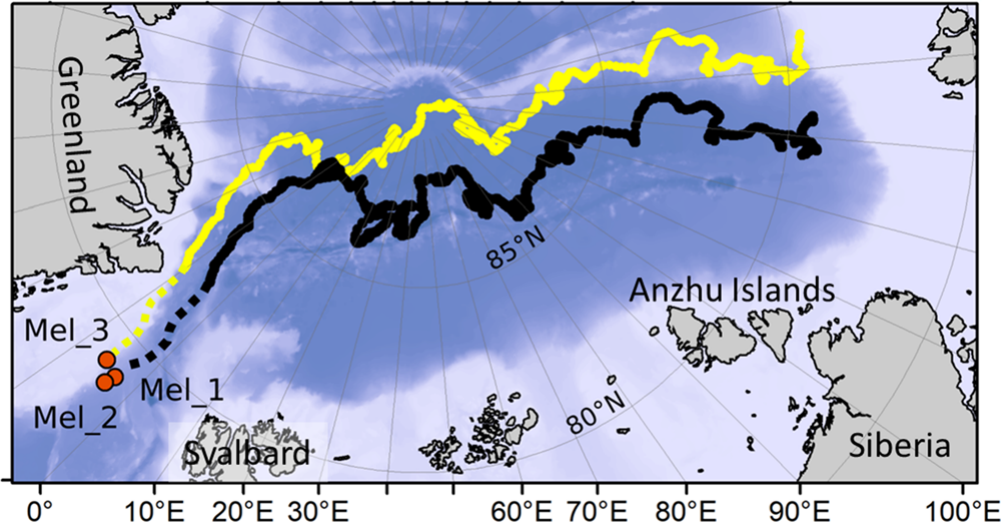
Drift trajectory of ice floes sampled pointing to formation in the Laptev Sea. Solid lines result from an automated sea ice tracking algorithm, while dashed lines are derived from a visual analysis of optical satellite data in the Fram Strait area.

There were statistical differences in MP concentrations for the MP count relative to wet weight (g) and volume (MP m^–3^) (Kruskal–Wallis one-way analysis of variance, df = 2, *H* = 8.436, and *p* = 0.0154) with significantly highest levels found in the easternmost sample Mel_1 (5.7 ± 1.7 × 10^4^ MP m^–3^), followed by the westernmost Mel_3 (2.2 × 10^4^ ± 8.1 × 10^3^ MP m^–3^) and nearby Mel_2 (1.3 × 10^4^ ± 8.7 × 10^3^ MP m^–3^, Figure 2A). Interestingly, the concentrations of MP in the sea water samples followed a similar trend to *M. arctica* samples from the respective sites with the highest levels found in Mel_1 (4.5 ± 1.5 × 10^3^ MP m^–3^) followed by Mel_3 (2.5 × 10^3^ ± 2.8 × 10^2^ MP m^–3^) and Mel_2 (1.4 ± 1.0 × 10^3^ MP m^–3^, Figure 2A). A pairwise *t*-test for independent means showed that *M. arctica* samples contained significantly higher MP levels compared with their respective sea water samples (*t*_(9)_ = −3.75, *p* = 0.006 for total sea water MP; *t*_(9)_ = 4.046, *p* = 0.003 for sea water MP < 25 μm). This could be interpreted as an indication of *M. arctica* trapping MPs from the surrounding sea and melt water during ice drift (Figure 3). While the water samples represent only snapshots in time, *M. arctica* could have trapped MPs from the surrounding water over time and thus accumulated MPs, leading to the higher concentrations measured. Interestingly, the sample Mel_2 with the lowest concentration floated near the remains of an ice berg (glacial origin, Figure 1A), whereas the other samples came from sea ice floes.

Since the ice berg (Mel_2) could not be located on high-resolution satellite data, suggesting that the source was unknown or that it drifted with the sea ice floes (Mel_1), it was combined with the latter’s drift trajectory (Figure 3). The paths of all ice floes sampled looked remarkably similar and suggest that the ice was 560 days old and formed off the Laptev Sea in mid-October (Figure 3). However, this origin does not necessarily reflect the origin of the ice algae. *M. arctica* can become detached due to strong currents and re-attach itself using extracellular polymeric substances.^15^ Therefore, this association could be quite dynamic. Still, the drift path taken is quite representative of sea ice in this area and could indicate where along the way MPs became entrained in the algal matrix. The MP concentrations in sea water (0–6.3 × 10^3^ MP m^–3^) were higher than previous measurements from the Fram Strait (0–1287 MP m^–3^),^9^ which is likely due to differences in the methodology used (32 μm pore size of pump filters compared to the 2.2 μm pore size limit in this study, μ-FTIR analysis, and larger water volume sampled in^9^). Constraining the sea water sample results to the particle size limit,^9^ our mean sea water samples for particles >25 μm (527 ± 294 MP m^–3^) are comparable to those previously published (421 MP m^–3^).

**Figure 4:**
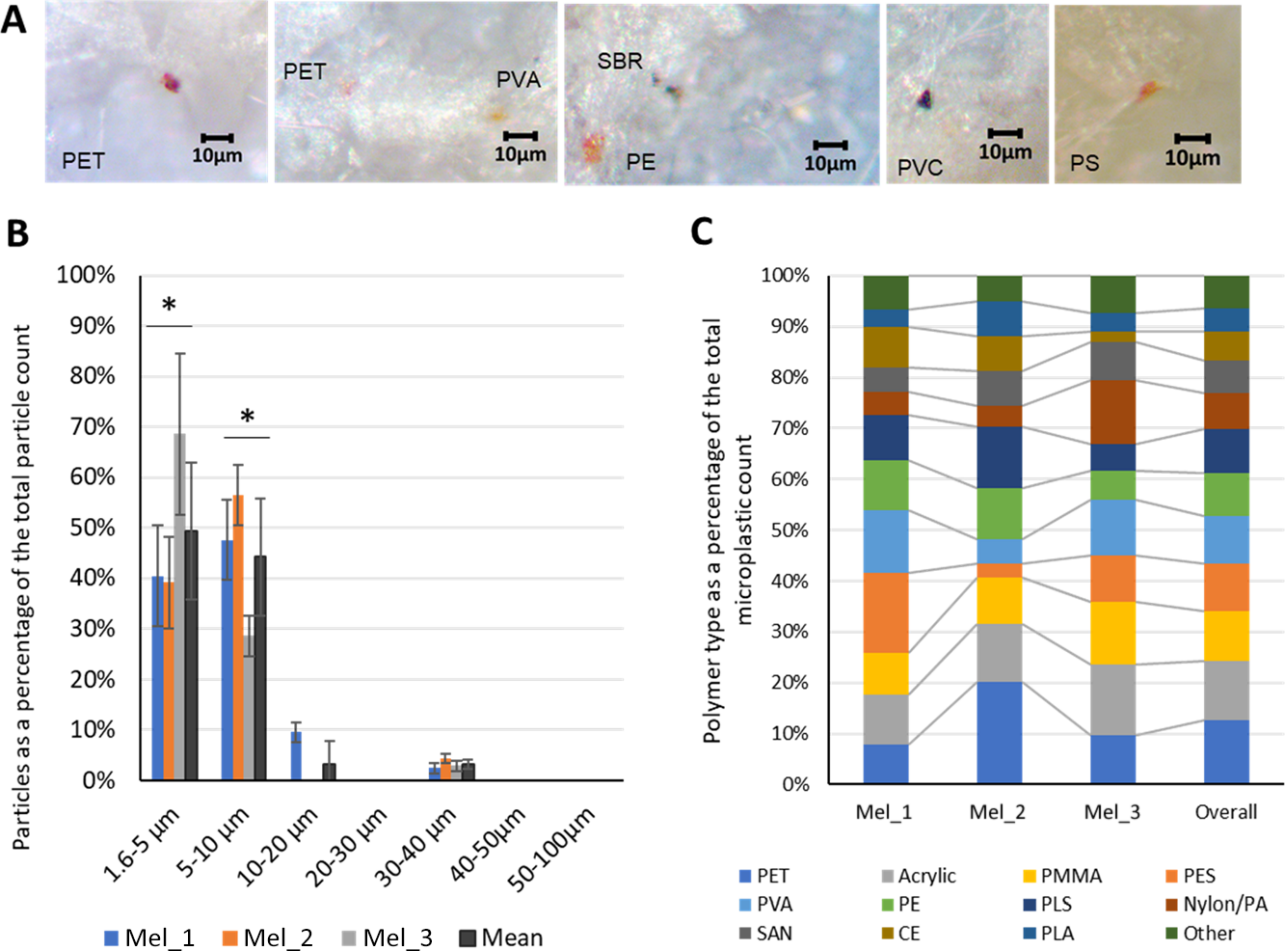
Characteristics of identified MPs. (A) Photographs of MPs. (B) Overarching and *M. arctica* sample MP particle size distribution. The asterisk indicates datasets that present a significant difference (* = *p* < 0.05). (C) Individual and overall polymer composition. Polymer composition within the samples varied but included polyethylene (PE), polyester (PES), polyethylene terephthalate (PET), polypropylene (PP), polystyrene (PS), polyvinyl chloride (PVC), polyurethane (PU), acrylonitrile butadiene styrene (ABS), polycarbonate (PC), polyethylene vinyl acetate (PVA), nylon, polyamide (PA), acrylic, cellulose acetate (CE), polymethyl methacrylate (PMMA), polysulfone (PLS), rubber, polylactic acid (PLA), styrene butadiene (SBT), styrene acrylonitrile copolymer (SAN), and others (<5%).

MP particles in both algal and sea water samples were generally smaller than 40 μm, with on average 94% (88–97%) of the total MP particles identified as 10 μm or smaller in size (Figure 4). Sample Mel_3, the most western site closest to the cold south-flowing Transpolar Drift current,^21^ presented a greater quantity of smaller MP particles (69% <5 μm), while the more eastern samples contained a slightly larger proportion of >5 μm particles (59–60%).

The *M. arctica* cell size ranges from 20 to 30 μm.^16,20^ Thus, these small MP particles could have been entangled in the filamentous threads, e.g., “stuck” to the outside of the algal aggregates, a process that could be helped by the mucous extracellular polymeric polysaccharides produced by the algae.^15,46^ Suspended MPs were also observed to adhere to the sticky surface of the seaweed *Fucus vesiculosus* in experiments.^47^ NP also adhered to green algae due to electrostatic attraction between NP and cellulose.^48^ What is more, adsorbed NP hindered algal photosynthesis in experiments, possibly by attenuating light,^48^ a process that has been reported for several algal species.^49^ Small MPs could also have occurred within the *M. arctica* cells. While differentiation was beyond the scope of this pilot study, it has been suggested that small MP particles could damage and enter algal cells, leading to oxidative stress responses including the damage of chloroplasts and thus inhibition of photosynthesis depending on the experimental doses used.^49^ Both mechanisms could have serious implications in terms of carbon sequestration and the ocean’s capacity to mitigate climate change, especially as MP concentrations are set to increase at the very least due to fragmentation of plastic debris that is already in the ocean.^3^

The occurrence and particle size distribution of these small MPs suggest that *M. arctica* could be entraining these smaller MP particles from the surrounding sea water and the melting sea ice. Sea ice is reported to contain MP particles up to 50 μm with a notable percentage of particles sized 11–25 μm,^7^ a larger range of particle sizes and a greater quantity of slightly larger particles than those found in the *M. arctica* samples. Similarly, snow and Arctic sea water are reported to present a larger MP range and particle size (up to 450 μm in snow and ≤200 μm in sea water).^9^

Despite the small sample size, the MP particles comprised 16 different polymer types without a clear dominance of any single polymer type (Figure 4). Similarly, Arctic sea ice, which is closely linked with ice algae, contained 17 different polymer types.^4^ PET accounted for the highest proportion of particles overall (13%). Polymers commonly used in paints and building/construction materials (acrylics and polymethyl methacrylate, PMMA) formed the second and third highest proportions of the particles (12 and 10%, respectively), concurring with elevated paint-related particles found in Arctic snow and water samples.^6,9^ Polyester (PES), polyethylene (PE), and polyamide (PA) were also notable in all samples (9, 9, and 7%, respectively, overall). Polyester and polyamide have been reported in Arctic sea water in high quantities (up to 39%).^8,9,50^ Similarly, polyvinyl chloride (PVC) was also reported in deep-sea sediments (1–13%),^51^ sea water (<30%),^50^ and sea ice (2–5%)^7,50^ from the Arctic. There is also an atmospheric component to the Arctic MP cycle,^5,10,52^ and atmospheric MPs are known to comprise a wide range of polymer types that can be rapidly transported over vast distances helped by resuspension of MPs from the sea surface.^25,53^ Both the polymer composition and the particle size distribution found in the *M. arctica* samples indicate that atmospheric deposition could play a role along with sea water transported into and out from the Arctic and from sea ice and snow melt.

### Possible Influence of Climate Change and *M. arctica* MP in the Arctic Food Chain

*M. arctica* is a primary producer that colonizes the underside of Arctic sea ice, growing during the summer periods when photosynthesis is possible due to available sunlight and nutrients.^54^ As the sea ice melts, the detached *M. arctica* is released into the water, where it produces high amounts of extracellular polymeric substances and forms aggregates.^46,55^ Such aggregates can stay afloat if irradiance promotes photosynthesis, and the resulting oxygen bubbles are captured in the viscous aggregates.^55^ During the next freeze-up, this could seed the new-forming ice and form the basis of new *M. arctica* colonies,^15^ a process that could also inoculate new-forming ice with MPs.

However, an important fraction of the *M. arctica* aggregates sinks to the Arctic seafloor, which accounted for 85% of the vertical carbon export in the Arctic in 2012.^16^ Katlein et al.^15^ suggested that aggregates with a diameter of 3 cm can reach the deep seafloor within a timeframe as short as 1 day. Bergmann et al.^5^ suggested that MPs bound to ice algal aggregates could contribute to the vertical export of MPs from the sea surface to the seafloor since concentrations of 6.3 × 10^6^ MP m^–3^ were measured in the deep-sea sediments beneath the marginal ice zone and a positive correlation with the chlorophyll *a* content was found.^9^

Following the current trend in climate change, resulting in hotter temperatures and more extreme weather events, sea ice will become thinner and less abundant^54,56^ and break up increasingly because of the increased storm frequency, potentially supporting greater ice algal blooms due to higher light availability. However, this is likely offset by nutrient limitation and the shorter frozen periods, with the ice habitat melting earlier and freezing up later than in past decades, resulting in shorter algal growth periods^57^ as well as lower biomass and deposition. If *M. arctica* is a key transport vector of MPs from the sea surface to the seafloor, then any shift in *M. arctica* growth and circulation will also modify the transport and distribution of MPs. However, other phytoplankton species, such as *Phaeocystis pouchetii*, which can form extensive floating gelatinous colonies in Arctic waters that also produce sticky exopolymer particles^58^ and sink to the seafloor,^59^ could play this role.

No matter what the consequences, it could be speculated that MPs from melting sea ice could be trapped by Arctic ice algae as a result of its filamentous and sticky morphology. Since ice algae are hotspots of biological activity and an important food source for grazing organisms,^14,19,60^ they could be a vector into under-ice food webs. Indeed, MPs were recently detected in grazing zooplankton from the Fram Strait.^11^ Similarly, it could pave the uptake of MPs by benthic organisms that have been observed to feed on ice algae deposited on the seafloor.^16,61^ While the effect of this uptake on Arctic species and ecosystems is not yet known,^5^ Arctic biota are already under serious pressure from global heating, which progresses four times faster in the Arctic compared with the globe.^62^ Plastic pollution likely exacerbates this pressure, so it needs to be tackled efficiently.^63^

## Supporting Information Available

MP counts in *M. arctica* and sea water samples from different locations; polymer types as percentage of the total MP count detected in *M. arctica* and sea water; particle size distribution of MPs in *M. arctica* samples; results of Kruskal–Wallis and *t*-tests and correlation and linear regression analyses; and results of positive control tests (PDF)
