# Supplementary material for: High Levels of Microplastics in the Arctic Sea Ice Alga Melosira arctica, a Vector to Ice-Associated and Benthic Food Webs
Source: Environ Sci Technol. 2023 Apr 21;57(17):6799–807. doi: 10.1021/acs.est.2c08010 (PMC10157888; doi:10.1021/acs.est.2c08010)
Supplement: Supplementary file 1 — es2c08010_si_001.pdf [file es2c08010_si_001.pdf]

Supporting Information for

High levels of microplastics in the Arctic sea ice alga *Melosira arctica*, a vector to ice-associated and benthic food webs

Melanie Bergmann, [1]*, Steve Allen, [2], Thomas Krumpen, [3], Deonie Allen, [4,5]

[1]*HGF-MPG Group for Deep Sea Ecology and Technology, Alfred-Wegener-Institut Helmholtz-Zentrum für Polar- und Meeresforschung (Melanie.Bergmann@awi.de)
[2]Ocean Frontiers Institute, Dalhousie University, Nova Scotia
[3]Sea Ice Physics, Alfred-Wegener-Institut Helmholtz-Zentrum für Polar- und Meeresforschung
[4]School of Geography, Earth and Environmental Science, University of Birmingham
[5]School of Physical and Chemical Sciences, University of Canterbury

This file includes:

- Table S1. MP counts detected in *Melosira arctica* samples.
- Table S2. MP counts detected in *Melosira arctica* and sea water samples as mean results representation for the sample location (MP m^{-3}).
- Table S3. MP polymer types as percentage of the total particle count detected in *Melosira arctica* and sea water.
- Table S4. Particle size distribution (in percentages per bin) for *Melosira arctica.*
- Note. S1. Kruskal–Wallis test is the one-way analysis of variance between *Melosira arctica* MP sample sites for MP relative to wet sample weight, dry sample weight and sample volume (MP m^{-3}).
- Note S2. t-Test of *Melosira arctica* individual samples to consider location specific differences and differences between *Melosira* and seawater samples.
- Note S3. Correlation and simple linear regression analysis of *Melosira* and sea water samples.
- Note S4. Positive control tests.

Table S1. MP counts detected in *Melosira arctica* samples. Sample counts are derived from µ-Raman spectra identification of MP particles.

Sample ID	PE	PES	PET	PP	PS	PVC	PU	ABS	PC	PVA	Nylon/PA	Acrylic	CE	PMMA	PLS	Rbr	PLA	SBR	SAN	Total MP Count	Total extrapolated filter count	Sample Wet Weight (g)	MP/g (wet weight)	Sample Dry Weight (g)	MP/g (dry weight)	Sample volume (m3)	MP/m3
MEL_1	5	8	5	0	2	1	0	0	1	6	4	7	3	5	4	0	2	0	1	54	216	3.3	66.1	0.61	355	0.002935	73593
MEL_1	3	5	0	0	1	0	0	0	0	4	0	2	3	2	3	0	1	0	2	26	104	3.3	31.8	0.61	171	0.002935	35435
MEL_1	3	5	5	0	1	0	0	0	0	4	2	2	3	2	3	0	1	1	2	34	136	3.3	41.6	0.61	223	0.002936	46319
MEL_1	5	8	4	0	2	1	0	0	1	6	3	7	3	5	4	0	2	1	2	54	216	3.3	66.2	0.61	355	0.002931	73701
MEL_2	6	3	6	0	0	2	0	1	1	4	2	6	2	4	7	0	2	0	2	48	192	7.7	25.0	0.58	330	0.006906	27803
MEL_2	1	0	5	0	0	1	0	0	0	0	1	1	1	1	1	0	1	0	1	14	56	7.7	7.3	0.58	96	0.006905	8110
MEL_2	1	0	2	0	0	0	0	0	0	0	0	1	1	1	1	0	1	0	1	9	36	7.7	4.7	0.58	62	0.006899	5218
MEL_2	2	1	2	0	0	1	0	0	0	2	1	3	1	2	3	0	1	0	1	20	80	7.7	10.4	0.58	138	0.006899	11595
MEL_3	3	4	6	1	2	0	1	0	0	3	5	6	2	7	2	1	1	1	2	47	188	7.2	26.0	0.57	328	0.0065	28921
MEL_3	1	2	0	0	0	0	0	0	0	3	0	3	0	2	1	0	1	0	2	15	60	7.2	8.3	0.57	105	0.0065	9230
MEL_3	3	4	5	1	2	0	1	0	0	4	4	6	2	7	3	0	1	1	3	47	188	7.2	26.0	0.57	328	0.006503	28911
MEL_3	1	2	5	1	0	1	0	0	0	3	10	3	0	2	1	0	1	0	2	32	128	7.2	17.7	0.57	224	0.006499	19695
blank_MEL_1	1	0	0	1	1	0	1	0	0	0	0	0	0	0	0	0	0	0	0	4							
blank_MEL_2	1	0	1	0	1	0	0	0	0	0	0	0	0	0	0	0	0	0	0	3							
blank_MEL_3	0	1	0	1	1	0	1	0	0	0	0	0	0	0	0	0	0	0	0	4							
blank_MEL_4	2	2	0	1	2	0	2	0	0	0	0	0	0	0	0	0	0	0	0	9							

Table S2. MP counts detected in *Melosira arctica* and sea water samples as mean results representation for the sample location (MP m^{-3}). Sample counts are derived from µ-Raman spectra identification of MP particles.

	MP/m3 (mean)	StDev	10th Percentile	90th Percentile
MEL_1	5.7E+04	1.7E+04	38700	73668
MEL_2	1.3E+04	8.7E+03	6086	22941
MEL_3	2.2E+04	8.1E+03	12370	28918
MEL Mean	3.1E+04	1.9E+04	8222	70865
SW_1	4.5E+03	1.5E+03	3030	5967
SW_2	1.4E+03	1.0E+03	264	2392
SW_2	2.5E+03	2.8E+02	2192	2727
SW Mean	2.8E+03	1.3E+03	1593	4095

Table S3. MP polymer types as percentage of the total particle count detected in *Melosira arctica* and sea water. Polymers identified include: polyethylene (PE), polyester (PES), polyethylene terephthalate (PET), polypropylene (PP), polystyrene (PS), polyvinyl chloride (PVC), polyurethane (PU), acrylonitrile butadiene styrene (ABS), polycarbonate (PC), polyethylene vinyl acetate (PVA), nylon, polyamide (PA), acrylic, cellulose acetate (CE), polymethyl methacrylate (PMMA), polysulfone (PLS), rubber, polylactic acid (PLA), styrene butadiene (SBT), styrene acrylonitrile copolymer (SAN).

Blank corrected *Melosira* Mean MP counts

Sample ID	PE	PES	PET	PP	PS	PVC	PU	ABS	PC	PVA	Nylon/PA	Acrylic	CE	PMMA	PLS	Rbr	PLA	SBR	SAN
MEL_1	10%	16%	8%	0%	4%	1%	0%	0%	1%	12%	5%	10%	8%	8%	9%	0%	4%	1%	5%
MEL_2	10%	3%	20%	0%	0%	4%	0%	1%	1%	5%	4%	11%	7%	9%	12%	0%	7%	0%	7%
MEL_3	6%	9%	10%	2%	2%	1%	1%	0%	0%	11%	13%	14%	2%	12%	5%	1%	4%	1%	8%
Overall	9%	9%	13%	1%	2%	2%	0%	0%	0%	9%	7%	12%	6%	10%	9%	0%	5%	1%	6%
StDev	3%	6%	9%	1%	2%	2%	1%	1%	1%	5%	8%	4%	4%	3%	4%	1%	2%	1%	3%
10th Percentile	6%	1%	1%	0%	0%	0%	0%	0%	0%	1%	0%	7%	0%	6%	4%	0%	2%	0%	4%
90th Percentile	11%	15%	22%	2%	4%	5%	2%	0%	2%	15%	10%	15%	11%	15%	14%	0%	7%	2%	11%

Blank corrected Sea Water Mean MP counts

Sample ID	PE	PES	PET	PP	PS	PVC	PU	ABS	PC	PVA	Nylon/PA	Acrylic	CE	PMMA	PLS	Rbr	PLA	SBR	SAN
SW1	0%	0%	6%	0%	18%	0%	4%	0%	0%	18%	0%	22%	0%	0%	10%	0%	22%	0%	0%
SW2	0%	28%	0%	0%	0%	33%	0%	0%	0%	0%	28%	0%	11%	0%	0%	0%	0%	0%	0%
SW3	0%	0%	11%	22%	0%	8%	11%	0%	0%	0%	0%	19%	19%	8%	0%	0%	0%	0%	0%
Overall	0%	9%	6%	7%	6%	14%	5%	0%	0%	6%	9%	14%	10%	3%	3%	0%	7%	0%	0%
StDev	0%	18%	11%	14%	9%	31%	11%	0%	0%	9%	18%	13%	15%	8%	6%	0%	11%	0%	0%
10th Percentile	0%	0%	0%	0%	0%	0%	0%	0%	0%	0%	0%	0%	0%	0%	0%	0%	0%	0%	0%
90th Percentile	0%	37%	20%	33%	18%	40%	17%	0%	0%	18%	37%	27%	33%	5%	13%	0%	25%	0%	0%

Table S4. Particle size distribution (in percentages per bin) for *Melosira arctica.* Particle size distribution values are derived using µ-Raman identification of polymers and Nile Red Fluorescence microscopy for morphology descriptions.

	MP polymer %			Standard Deviation						
Particle size (count)	MEL_1	MEL_2	MEL_3	MEL_1	MEL_2	MEL_3	Overall Mean	St Dev	10th Percentile	90th Percentile
1.6-2µm	7%	4%	31%	8%	12%	14%	14%	12%	5%	27%
2-5 µm	33%	35%	37%	3%	2%	3%	35%	2%	34%	37%
1.6-5 µm	40%	39%	69%	10%	9%	16%	49%	14%	39%	63%
5-10 µm	48%	57%	29%	8%	6%	4%	44%	12%	32%	55%
10-20 µm	10%	0%	0%	2%	0%	0%	3%	4%	0%	8%
20-30 µm	0%	0%	0%	0%	0%	0%	0%	0%	0%	0%
30-40 µm	2%	4%	3%	1%	1%	1%	3%	1%	2%	4%
40-50µm	0%	0%	0%	0%	0%	0%	0%	0%	0%	0%
50-100µm	0%	0%	0%	0%	0%	0%	0%	0%	0%	0%
100-150µm	0%	0%	0%	0%	0%	0%	0%	0%	0%	0%
150-200µm	0%	0%	0%	0%	0%	0%	0%	0%	0%	0%
200-250µm	0%	0%	0%	0%	0%	0%	0%	0%	0%	0%
250-300µm	0%	0%	0%	0%	0%	0%	0%	0%	0%	0%
300-350µm	0%	0%	0%	0%	0%	0%	0%	0%	0%	0%
350-400µm	0%	0%	0%	0%	0%	0%	0%	0%	0%	0%
400-450µm	0%	0%	0%	0%	0%	0%	0%	0%	0%	0%

450-500µm	0%	0%	0%	0%	0%	0%	0%	0%	0%	0%
500-550µm	0%	0%	0%	0%	0%	0%	0%	0%	0%	0%
550-600µm	0%	0%	0%	0%	0%	0%	0%	0%	0%	0%
600-650µm	0%	0%	0%	0%	0%	0%	0%	0%	0%	0%
650-700µm	0%	0%	0%	0%	0%	0%	0%	0%	0%	0%
700-750µm	0%	0%	0%	0%	0%	0%	0%	0%	0%	0%
750-800µm	0%	0%	0%	0%	0%	0%	0%	0%	0%	0%
800-850µm	0%	0%	0%	0%	0%	0%	0%	0%	0%	0%
850-900µm	0%	0%	0%	0%	0%	0%	0%	0%	0%	0%
900-950µm	0%	0%	0%	0%	0%	0%	0%	0%	0%	0%
950-1000µm	0%	0%	0%	0%	0%	0%	0%	0%	0%	0%
1000-2000µm	0%	0%	0%	0%	0%	0%	0%	0%	0%	0%
2000-3000µm	0%	0%	0%	0%	0%	0%	0%	0%	0%	0%
3000-4000µm	0%	0%	0%	0%	0%	0%	0%	0%	0%	0%
4000-5000µm	0%	0%	0%	0%	0%	0%	0%	0%	0%	0%
Percentage fibres	1%	3%	4%							
Percentage fibres in blanks (mean)	13%									

Note. S1. Kruskal–Wallis test is the one-way analysis of variance between *Melosira arctica* MP sample sites for MP relative to wet sample weight, dry sample weight and sample volume (MP m^{-3}). This one-factor, non-parametric ANOVA evaluates the differences between multiple independent sample sets based on ranks. The Kruskal-Wallis test is calculated following:

$$\boldsymbol{H} = \frac{12}{n(n+1)} \sum \left(\frac{{R_j}^2}{n_j} \right) - 3(n+1) \qquad \text{Eqn. 1}$$

Where:
H = hypothesis
n = total sample size across all groups
n_j = sample size of group j
R_j = rank sum of group j

**Total extrapolated filter count**	**MP/g (wet weight)**	**MP/g (dry weight)**	**MP/m3**
H = 3.115 < 5.692	H=8.346≥5.692	H=2.8077 < 5.692	H=8.3462 < 5.692
p-value is 0.2083 > 0.05 Ho is not rejected. Therefore, there is not enough evidence to claim that not all population medians are equal, at the 0.05 significance level.	p-value is 0.0154 < 0.05 Ho is rejected. Therefore, there is enough evidence to claim that not all population medians are equal, at the 0.05 significance level.	p-value is 0.2457 > 0.05 H0 cannot be rejected. the difference between the mean ranks of all groups is not big enough to be statistically significant	p-value is 0.0154 > 0.05 H0 is rejected. The difference between the mean ranks of some groups is big enough to be statistically significant.

R (region of acceptance) ={H≥5.692}

Note S2. t-Test of *Melosira arctica* individual samples to consider location specific differences and differences between *Melosira* and seawater samples. t-Test analysis is used to help consider if there is a significant difference between sample location MP results, following:

$$\boldsymbol{t} = \frac{\bar{x}_1 - \bar{x}_2}{\sqrt{\left(s^2\left(\frac{1}{n_1}+\frac{1}{n_2}\right)\right)}} \qquad \text{Eqn. 2}$$

Where:
t = t-value
n = number of observations in the group
x = means of the groups

Mean of Mel vs Mean of SW

t-test	t-Test: Paired Two Sample for Means	t-Test: Two-Sample Assuming Equal Variances
t(3) =	2.217092	2.064296
p value =	0.078457	0.053963

t-test	Total Mel vs total SW	Total Mel vs total SW <25µm

t(9) =	-3.75239	4.04634
p value =	0.00627	0.00315

t-test	Mel_1+SW_1 vs Mel_2+SW_2	Mel_1+SW_1 vs Mel_3+SW_3	Mel_2+SW_2 vs Mel_3+SW_3
t(6) =	2.36515	1.66468	-1.11649
p value =	0.017122	0.060924	0.142211

t-test	Mel1 wet vs dry	Mel2 wet vs dry	Mel3 wet vs dry	Mel total wet vs dry
t(3) =	-4.71867	-2.4069	-4.2471	-6.11734
p value =	0.001631	0.026398	0.002699	< 0.0001

Note S3. Correlation and simple linear regression analysis of *Melosira* and sea water samples.

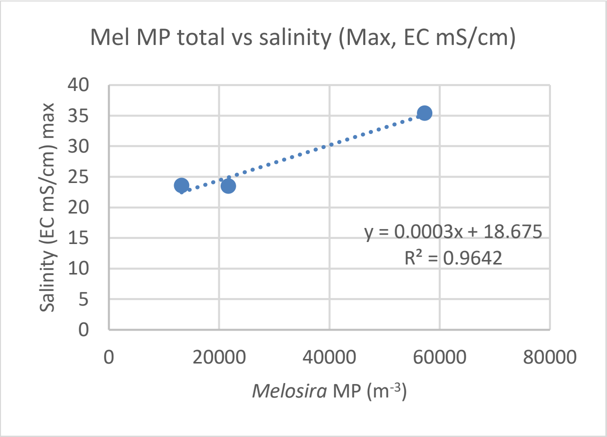

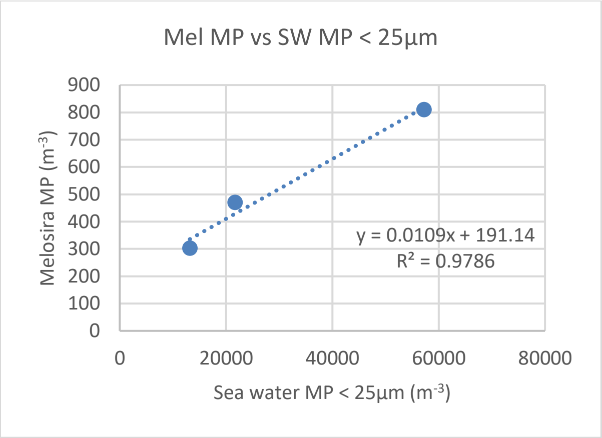

	Melosira MP (m^{-3})				Sea Water MP (m^{-3})					
	<10µm	<20µm	1.6-5 µm	5-10µm	Total	MP>25µm	MP<25µm	Ice thickness (m)	Salinity (EC mS/cm) min	Salinity (EC mS/cm) max
One-tailed										
t(2) =	2.28	2.28	2.25	2.28	2.06	2.10	2.24	2.28	2.27	2.27
p =	0.04	0.04	0.43	0.43	0.05	0.05	0.04	0.04	0.04	0.04
Two-tailed										
t(2) =	2.28	2.28	2.28	2.28	2.06	2.10	2.24	2.28	2.27	2.27
p =	0.09	0.09	0.85	0.09	0.11	0.10	0.09	0.09	0.09	0.09
r =	-0.94	0.81	-0.30	0.02	0.99	0.98	0.99	0.49	-0.30	0.98
R^2 =	0.89	0.65	0.09	0.00	0.97	0.97	0.98	0.24	0.09	0.96

Note S4. Positive control tests.

	Particle count	Expected #	Analytical efficiency
Positive control 1	33	34	97%
Positive control 2	59	67	88%
Positive control 3	93	134	69%
Positive control 4	25	25	100%
Average			89%
Standard deviation			12%
Positive control 5	284	296	96%
Positive control 6	565	593	95%
Positive control 7	82	111	74%
Positive control 8	77	111	69%
Positive control 9	24	25	96%
Average			86%
Standard deviation			12%

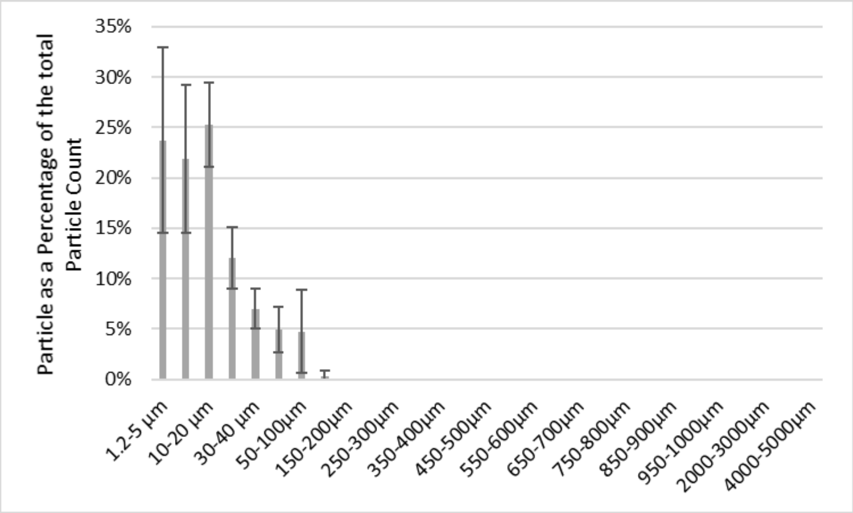

Particle size distribution of the control MP spike particles (polyethylene fragments, non-spherical).
